# Nanobiomechanics in Oncology: Atomic Force Microscopy for Cancer Mechanophenotyping Using Cellular Young's Modulus

**DOI:** 10.1002/snz2.70090

**Published:** 2026-10-29

**Authors:** Hermawan Tjoa, Li Mei, Harsha De Silva, Darryl Tong, Guangzhao Guan

**Affiliations:** ^1^ Department of Oral Diagnostic and Surgical Sciences Faculty of Dentistry University of Otago Dunedin New Zealand; ^2^ Department of Oral Sciences Faculty of Dentistry University of Otago Dunedin New Zealand

**Keywords:** atomic force microscopy (AFM), cancer, Young's modulus

## Abstract

This review examines the current literature on the application of atomic force microscopy (AFM) to cancer mechanophenotyping, with particular emphasis on cellular Young's modulus and its potential diagnostic significance. A structured electronic search of PubMed, Scopus and Google Scholar was conducted to identify studies published between 1999 and 2025 reporting AFM‐based measurements of cellular Young's modulus in cancer‐related models. Across multiple malignancies, reduced cellular stiffness is frequently observed in malignant compared with non‐malignant cells and is associated with cytoskeletal remodelling, increased deformability and altered cell–matrix interactions. However, the relationship between Young's modulus and malignancy is not universally monotonic. Nanomechanical properties may vary across different pathological states and malignant phenotypes rather than following a simple monotonic trajectory, while invasive and metastatic cells can dynamically remodel their cytoskeleton and modify their stiffness in response to environmental and mechanical constraints. Young's modulus should therefore not be regarded as an isolated universal cancer biomarker, but rather as one component of a dynamic mechanophenotype influenced by disease stage, cellular region, microenvironment and measurement methodology. Recent advances in automated force acquisition, optical cell recognition, artificial intelligence and high‐throughput AFM are beginning to address traditional limitations in measurement efficiency and operator dependence. The future diagnostic value of AFM may therefore lie in integrating Young's modulus with complementary parameters, including spatial mechanical heterogeneity, viscoelasticity, adhesion, deformation‐energy characteristics and nanotopography, to define multidimensional cancer mechanophenotypes. Standardised acquisition and analysis protocols, together with validation in patient‐derived specimens, will be essential for translating AFM nanomechanics from an experimental measurement into a clinically informative biomechanical biomarker.

## 1 Introduction

Cancer remains a major global health burden, accounting for approximately one in six deaths worldwide (World Health Organization 2026). Prognosis is strongly influenced by the stage at diagnosis, with metastatic disease being responsible for most cancer‐related mortality (Eccles and Welch 2007; World Health Organization 2026). Earlier identification of malignant transformation is therefore critical to improving treatment outcomes. Histopathological examination of tissue remains the gold standard for cancer diagnosis and grading and provides important prognostic information (He et al. 2012), often supplemented by techniques such as immunohistochemistry (Oliveira and Ribeiro‐Silva 2011; Selves et al. 2018). However, these approaches generally require tissue biopsy and primarily assess morphological and molecular abnormalities that are already detectable within the sampled tissue. There is consequently considerable interest in identifying complementary biomarkers capable of detecting earlier cellular changes associated with carcinogenesis.

### 1.1 Cell Biomechanics and Cancer

The mechanical properties of cells are increasingly recognised as potential biomarkers of malignant transformation (Suresh 2007; Zhou et al. 2025). These properties are governed to a large extent by the cytoskeleton and its mechanical coupling to the extracellular matrix (ECM) (Suresh 2007). The cytoskeleton comprises actin filaments, intermediate filaments and microtubules, which collectively determine the cell shape and mechanical integrity and participate in processes including adhesion, migration, proliferation and division (Fletcher and Mullins 2010; Yamazaki et al. 2005). Carcinogenesis is accompanied by extensive remodelling of this architecture. In particular, reorganisation of the actin cytoskeleton, altered cell–cell and cell‐ECM adhesion and epithelial–mesenchymal transition (EMT) promote cellular motility and invasive behaviour, while alterations in cytoskeletal organisation are also associated with changes in cellular deformability (Radisky 2005; Suresh 2007; Tomaskovic‐Crook et al. 2009; Yamazaki et al. 2005).

These structural alterations are reflected in measurable changes in cellular mechanical properties, including elasticity, viscoelasticity, adhesion and deformability (Suresh 2007). Among these, cellular stiffness or elasticity has been particularly widely investigated (Zhou et al. 2025). Young's modulus is commonly used as an apparent measure of cellular stiffness, relating applied mechanical loading to the resulting deformation within an assumed elastic model. However, living cells are intrinsically viscoelastic, heterogeneous and dynamically remodelling materials rather than ideal elastic solids (Eliahoo et al. 2024; Zhou et al. 2025). Consequently, Young's modulus obtained from cellular measurements is dependent on the measurement technique, probing scale, loading rate, indentation depth and modelling assumptions and is most appropriately interpreted as an apparent mechanical parameter for comparisons made under defined experimental conditions.

Across many cancer models, malignant cells have been reported to exhibit a lower apparent Young's modulus and greater deformability than their non‐malignant counterparts, although exceptions have been described (Nikkhah et al. 2010; Zemła et al. 2018). This mechanical softening is closely associated with cytoskeletal remodelling, particularly altered organisation and polymerisation of the actin network and may facilitate the deformation required for tumour cells to migrate through the ECM and participate in the metastatic cascade (Lee and Lim 2007; Suresh 2007; Zemła et al. 2018). Metastatic dissemination requires tumour cells to detach and invade surrounding tissues, intravasate into the circulation and subsequently extravasate into distant sites (Di Russo et al. 2024). Importantly, emerging evidence suggests that cellular mechanical properties may vary across different pathological states and malignant phenotypes rather than differing only between fully malignant and normal cells (Zemła et al. 2018). Consequently, cellular mechanics may provide information not only about the presence of malignancy but also about its differences among pathological states and malignant phenotypes.

### 1.2 Atomic Force Microscopy (AFM)

AFM provides a means of interrogating these mechanical alterations at the single‐cell level. AFM measures the interaction between a nanoscale probe mounted on a flexible cantilever and the sample surface (Figure 1). During force spectroscopy, the probe indents the cell under a controlled load, generating a force–distance curve (Figure 2) that can be analysed using contact‐mechanics models, such as the Hertz or Sneddon models, to estimate Young's modulus (Gavara 2017; Guan et al. 2025; Kuznetsova et al. 2007). This enables quantitative assessment of cellular stiffness with high spatial resolution under near‐physiological conditions (Deng et al. 2018).

**FIGURE 1:**
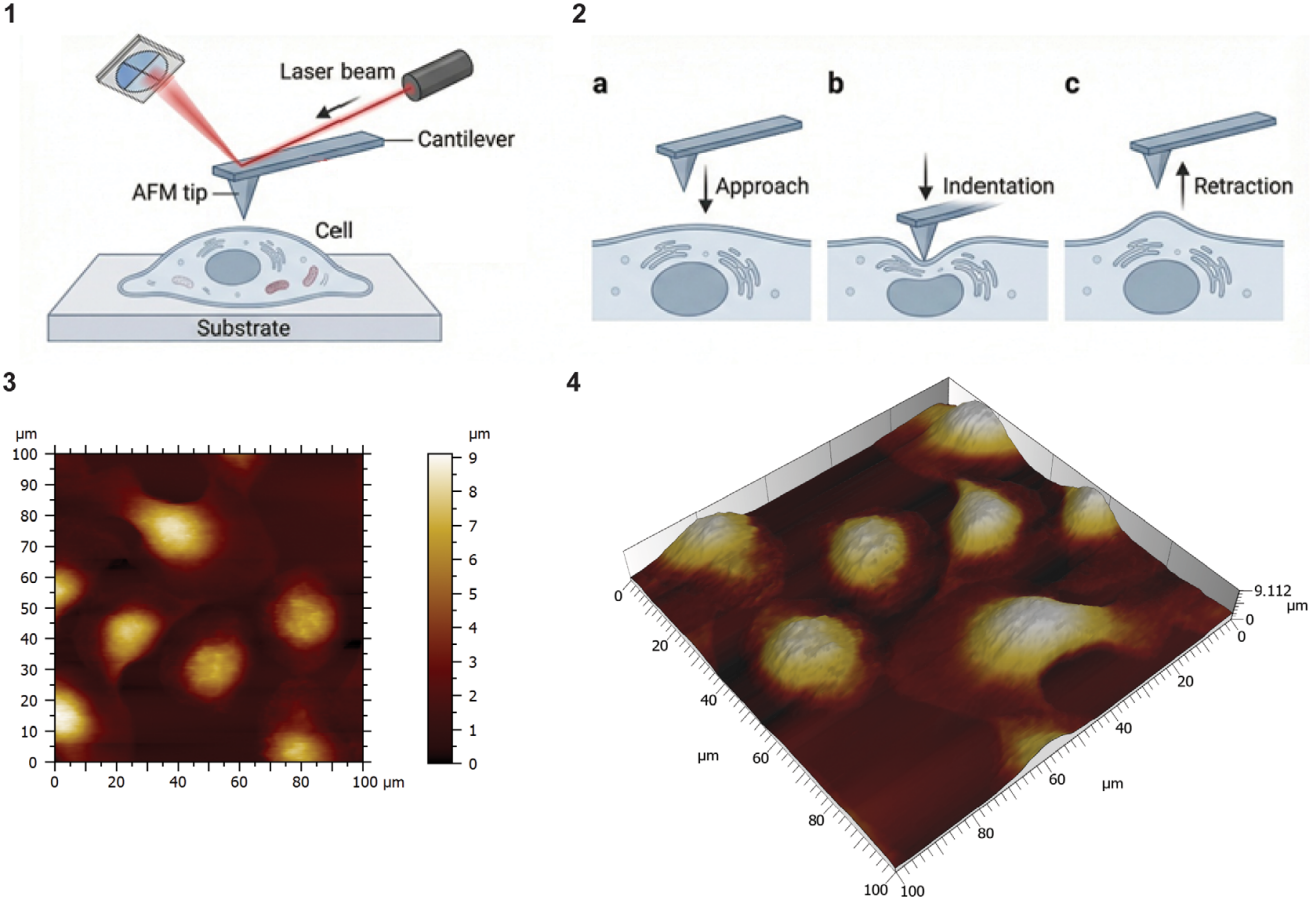
Schematic illustration of AFM. (1) and (2) Interaction of the AFM tip with the sample/cell causes cantilever deflection, which is detected as a change in the laser signal at the photodiode (created with BioRender.com). (3) and (4) 2D and 3D representations for cells.

**FIGURE 2:**
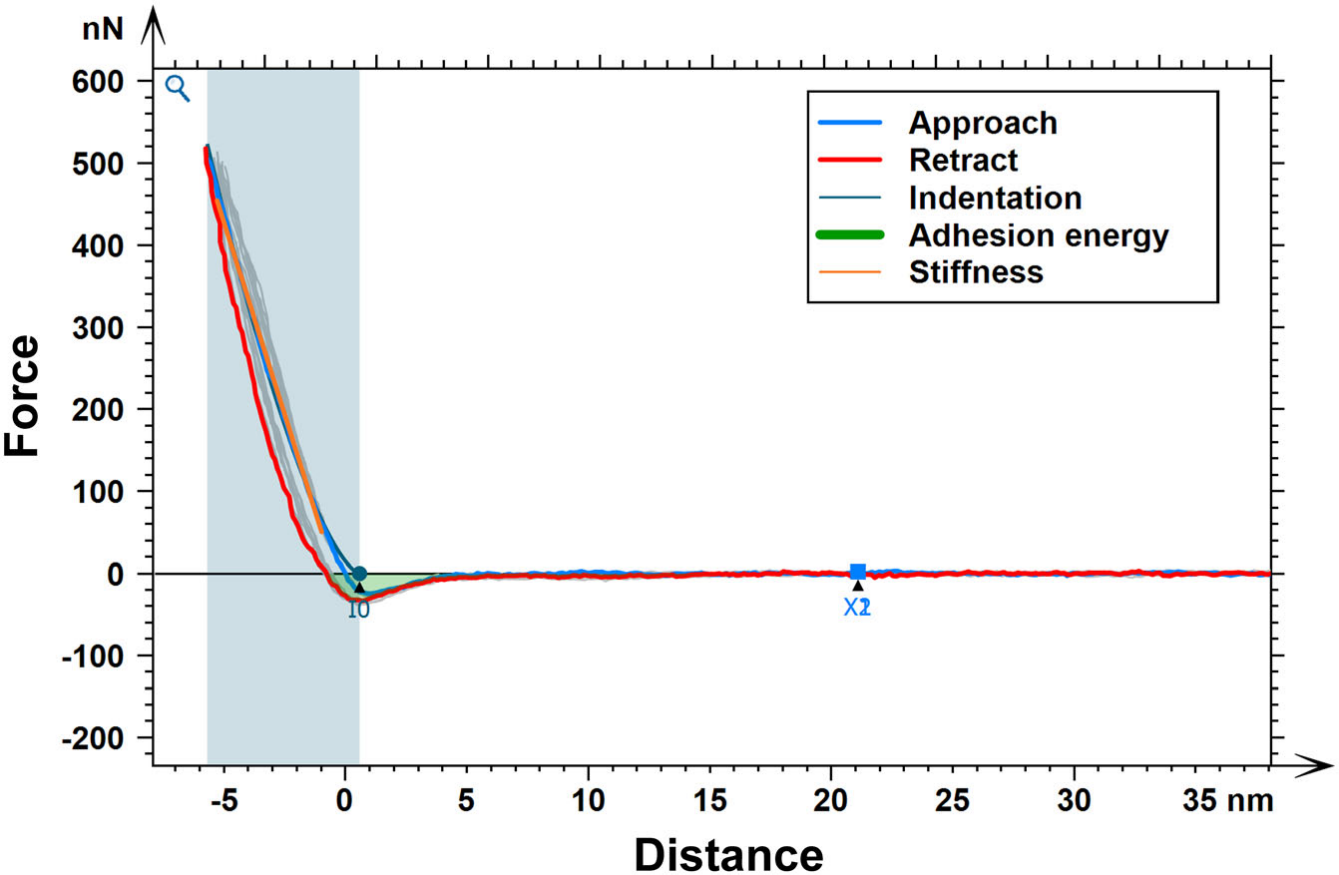
Schematic AFM force–distance curve. The curve represents the interaction between the AFM tip and the sample surface during approach and retraction. On the *x*‐axis is the tip–sample separation distance, and on the *y*‐axis is the force applied to the cantilever. During the approach, the tip initially experiences no force, followed by a small attractive regime (negative force) as van der Waals and other interactions pull the tip towards the surface. Upon contact, the tip exerts a repulsive force on the sample, with the slope indicating sample stiffness. During retraction, adhesive interactions can temporarily pull the tip towards the surface, producing an adhesion peak (maximum negative force) before the force returns to zero. Key features, including the contact point, repulsive regime, attractive regime and adhesion force, are labelled, illustrating how AFM force–distance curves can be used to measure mechanical properties such as cellular stiffness (Young's modulus) and surface adhesion.

At a given indentation depth, cells with a higher apparent Young's modulus generally exhibit greater resistance to deformation. However, conventional contact‐mechanics models employ idealised assumptions of elastic, homogeneous contact, whereas living cells are heterogeneous, viscoelastic and of finite thickness (Gavara 2017; Guz et al. 2014). Consequently, the estimated modulus is influenced by probe geometry, indentation depth, loading rate, contact‐point determination, measurement location, substrate effects and the particular mechanical model applied (Gavara 2017; Kuznetsova et al. 2007). Furthermore, the cell surface is covered by a pericellular brush layer comprising glycocalyx components and membrane protrusions such as microvilli and microridges, which contributes to the initial interaction between the AFM probe and the cell surface (Iyer et al. 2009). Failure to account for this non‐Hertzian contribution may complicate identification of the effective contact interface and produce indentation‐depth‐dependent estimates of Young's modulus (Guz et al. 2014; Makarova and Sokolov 2022). Brush‐aware analysis attempts to separate the mechanical contribution of the pericellular layer from deformation of the underlying cell body and can improve the self‐consistency of effective cell‐body modulus measurements, particularly when relatively blunt probes are used (Guz et al. 2014; Makarova and Sokolov 2022).

In addition to measuring Young's modulus, AFM can map spatial variations in mechanical properties across individual cells and generate high‐resolution nanotopographic images (Figure 1) (Gavara 2017; Kuznetsova et al. 2007). Conventional AFM topography primarily characterises cell‐surface morphology, whereas force spectroscopy and force mapping characterise local mechanical responses. Spatial variations in stiffness may correlate with underlying cytoskeletal organisation, including changes in the actin architecture, and cytoskeletal disruption has been associated with altered cellular deformability and mechanical behaviour (Kuznetsova et al. 2007; Nikkhah et al. 2010; Zemła et al. 2018). However, conventional AFM measurements do not independently identify specific intracellular structures such as filamentous actin (F‐actin), microtubules or intermediate filaments in intact living cells. Attribution of AFM‐derived topographical or mechanical features to particular cytoskeletal components therefore requires complementary biological validation, such as fluorescence microscopy, immunostaining or targeted cytoskeletal perturbation (Gavara 2017; Nikkhah et al. 2010). AFM thus provides spatially resolved information on cellular morphology and mechanical heterogeneity, but mechanical contrasts should not in themselves be interpreted as direct measurements of specific cytoskeletal structures.

Among the biomechanical parameters measurable by AFM, Young's modulus remains among the most extensively investigated in cancer mechanobiology and provides an experimentally accessible measure of cellular resistance to deformation. Its biological relevance arises in part from its sensitivity to cytoskeletal and cortical organisation; however, it should not be interpreted as a direct or invariant measure of cytoskeletal integrity. Rather, AFM‐derived Young's modulus should be regarded as an apparent or effective mechanical parameter whose magnitude depends on both the biological state and experimental conditions (Guz et al. 2014; Zemła et al. 2018). Probe geometry, indentation depth, loading rate and force, contact‐point determination, cellular region, cell morphology, cell–substrate interactions and the selected contact‐mechanics model can all influence the resulting value (Deng et al. 2018; Gavara 2017; Guz et al. 2014; Zemła et al. 2018). This methodological dependence can produce substantial variation in reported cellular stiffness. Indeed, comparative measurements of MCF‐7 cells using different mechanical techniques demonstrated differences in elastic modulus spanning several orders of magnitude, reflecting differences in applied stress, deformation rate, probe geometry, measurement location and extracellular environment (Wu et al. 2018). Young's modulus is therefore most informative when interpreted as a context‐dependent component of a broader cellular mechanophenotype rather than as an isolated intrinsic material constant, and comparisons across cancer types, disease stages, laboratories and experimental platforms require appropriate methodological context and standardisation.

Previous reviews have established AFM‐based cancer mechanobiology as a well‐developed field. Alibert et al. (2017) critically examined whether cancer cells are consistently softer than their normal counterparts and highlighted the context dependence of cellular mechanical phenotypes. Lekka (2016) summarised AFM studies comparing the mechanical properties of normal and cancerous cells and emphasised the relative nature of Young's modulus measurements, while Zemła et al. (2018) reviewed AFM‐based characterisation of cancer cells and tissues, encompassing cellular elasticity, deformability and adhesive interactions. More recent reviews and methodological studies have broadened this scope to encompass cellular and tissue morphology, surface nanotopography and roughness, viscoelasticity, tumour microenvironment interactions, treatment response and emerging computational approaches to AFM‐based classification (Deng et al. 2018; Guan et al. 2025; Zhou et al. 2025).

Building on this literature, the present review critically evaluates AFM‐derived Young's modulus across cancer‐related pathological states and malignant phenotypes. Rather than considering cellular softening as a universal endpoint, we examine Young's modulus within a dynamic mechanobiological continuum encompassing premalignant transformation, spatial heterogeneity, cytoskeletal remodelling, invasion and metastatic mechanical plasticity. We further consider how Young's modulus can be integrated with complementary AFM‐derived parameters, including viscoelasticity, adhesion, deformation energy and nanotopography and evaluate recent advances in higher‐throughput and computational AFM analysis that may facilitate clinical translation. In this context, we advocate a shift from single‐parameter stiffness measurement towards multidimensional AFM‐based cancer mechanophenotyping, with emphasis on biological interpretation, methodological standardisation and clinical validation.

## 2 Literature Search and Selection Methods

The aim of this narrative review was to critically evaluate the literature on the use of AFM to characterise Young's modulus in cancer‐related cellular and tissue models, with particular emphasis on its potential diagnostic and translational significance. A structured electronic literature search was conducted using PubMed, Scopus and Google Scholar to identify relevant studies published between 1999 and 2025. Searches used combinations of controlled vocabulary, where applicable and free‐text terms including ‘AFM', ‘neoplasm', ‘cancer', ‘elastic modulus', Young's modulus' and cell biomechanics;. Reference lists of relevant primary studies and review articles were also manually screened to identify additional eligible publications.

Studies were included if they reported AFM‐based measurements of Young's modulus in human cancer‐related models and compared mechanical properties between non‐malignant and malignant cells or tissues, or across different pathological stages, histological grades or metastatic phenotypes. Review articles, non‐human studies, non‐English‐language publications and studies that did not report AFM‐derived Young's modulus were excluded from the primary study synthesis. Review articles were nevertheless used to provide background context and to identify additional primary studies. Figure 3 summarises the literature search and study‐selection process.

**FIGURE 3:**
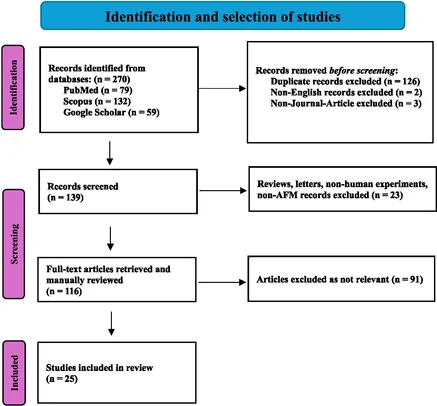
Literature search and study‐selection flow diagram.

## 3 Results

Table 1 summarises the included AFM studies reporting Young's modulus in cancer‐related models.

**TABLE 1:** Summary of included AFM studies reporting Young's modulus in cancer‐related models.

Neoplasm	Samples	AFM/key measurement conditions	Young's modulus/main findings	References [Author (year), Country]
Breast				
	Epithelium: • Benign: MCF‐10A • Malignant: MCF‐7	Nanoscope IV 4.5‐μm spherical probe (*k* ≈ 0.01 N/m); live cells at 37 °C; central‐cell indentation at 0.03–1 Hz with 200 pN force; Hertz model, *ν* = 0.5	MCF‐10A approximately 1.4–1.8× stiffer than MCF‐7 Absolute values not reported as a single fixed value because modulus varied with loading rate	Li et al. (2008), Singapore
	Epithelium: • Benign: MCF‐10A • Malignant: MCF‐7	JPK NanoWizard III, Germany MLCT cantilever (*k* ≈ 0.01 N/m); live cells in HBSS at 37 °C; central‐cell indentation at ∼2 μm/s with 1 nN force; Hertz model	MCF‐10A: 1.231 ± 0.755 kPa MCF‐7: 0.487 ± 0.173 kPa In addition to being significantly softer, MCF‐7 also demonstrated lower viscosity, consistent with a softer and more fluid‐like mechanical phenotype	Wang et al. (2016), China
	MCF‐10A (normal) MCF‐7 (cancerous)	BioScope Catalyst, USA Central cellular region; 8 µm/s loading rate; 5 nN maximum force; approach‐reside‐retract protocol; Sneddon fitting	MCF‐10A: 5.03 ± 0.16 kPa MCF‐7: 3.57 ± 0.15 kPa MCF‐7 is 1.4x less stiff than MCF‐10A. Relaxation time (representing viscoelasticity) significantly less for MCF‐7	Li et al. (2018), China
	MDA‐MB‐231 (metastatic breast cancer cell line)	Asylum Research, UK Combined AFM–confocal microscopy; sphero‐conical probe (∼700 nm radius); MDA‐MB‐231 cells invading collagen‐I hydrogels; substrate‐corrected sphero‐conical model with finite‐element validation	Median Young's modulus embedded within collagen I hydrogels 0.74 ± 0.29 kPa (0%–50% embedded) 0.99 ± 0.38 kPa (50%–75% embedded) 1.36 ± 0.50 kPa (75%–100% embedded) 1.5 ± 0.40 kPa (fully embedded) Invading/embedded MDA‐MB‐231 cells were significantly stiffer than cells remaining on top of collagen‐I. ROCK inhibition reduced this stiffening, supporting an actomyosin‐contractility contribution	Staunton et al. (2016), USA
	Breast cancer sample tissues from biopsy and sample of normal tissues surrounding the tumours	JPK NanoWizard III, Germany Conical probe (*k* ≈ 0.15 N/m); fresh breast tissue in DMEM at 37 °C; 1.5 nN loading force; modified Hertz model, *ν* = 0.5; C‐means clustering used to identify putative cellular, intermediate and fibrous regions	Young's modulus by region Cellular: normal 1.427 kPa, cancer 0.709 kPa Intermediate: normal 5.487 kPa, cancer 5.199 kPa Fibrous: normal: 14.456, cancer 16.055 kPa The mean modulus of the cellular region was ∼50% lower in cancerous than healthy tissue (*p* < 0.05). Conversely, cancerous‐tissue ECM was ∼11% stiffer than healthy ECM, but this was not significant	Ansardamavandi et al. (2016), Iran
	Cancer cells • Non‐metastatic: MCF‐7 • EGF‐treated MCF‐7 • Highly metastatic: MDA‐MB‐231 Epidermal growth factor (EGF) drives cancer cells across the epithelial‐mesenchymal transition (EMT)	JPK/Bruker, Germany QI nanomechanical mapping with sharp PF‐QNM probe (∼65 nm radius; *k* ≈ 0.06–0.1 N/m); free cells on fibronectin‐coated substrate or geometrically constrained on Y‐shaped fibronectin micropatterns; 0.3 nN at 100 μm/s; Sneddon model	MCF‐7: 4.7 ± 2.1 kPa EGF‐treated MCF‐7: 3.8 ± 1.7 kPa MDA‐MB‐231: 3.5 ± 2.0 kPa Young's modulus of MCF‐7 higher in the periphery than in the centre; such differences less noticeable in EGF‐treated MCF‐7 and MDA‐MB‐231 When cell geometry was constrained on Y‐shaped fibronectin micropatterns, MCF‐7 was ∼4.0 ± 1.5 kPa, EGF‐treated MCF‐7 softened markedly to ∼1.3 ± 0.4 kPa, whereas MDA‐MB‐231 stiffened to ∼7.3 ± 4.8 kPa and actually became stiffer than MCF‐7 F‐actin MCF‐7: denser & more concentrated in the periphery than in the centre MDA‐MB‐231: disorganised and sparse F‐actin across the cell	Liboz et al. (2023), France
	Breast adenocarcinoma MDA‐MB‐435 • 435/BRMS1 • 435 Breast cancer metastasis suppressor 1 (BRMS1) gene suppresses metastasis. The cell line 435/BRMS1 is MDA‐MB‐435 transfected with BRMS1	PicoPlus, USA Contact‐mode AFM in culture medium; force–distance curves used to determine adhesion, cellular spring constant and Young's modulus; Sneddon cone model	435/BRMS1: 60.36 ± 31.42 kPa 435: 4.55 ± 2.04 kPa BRMS1 expression was associated with increased Young's modulus, cell spring constant and surface adhesion, together with cytoskeletal reorganisation	McEwen et al. (2013), USA
	Cancer cell lines • MCF‐7 (non‐invasive) • T47D (slightly invasive) • MDA‐MB‐231 (highly invasive)	JPK Instruments, Germany AFM indentation: tetrahedral probe (*k* ≈ 0.04 N/m), ≤1 nN force and ≤0.5 μm indentation over the nucleus at 37 °C; Hertz model. SCFS: cell–cell contact at 2.5 nN for 10 or 120 s; detachment force measured on retraction	MCF‐7: 1.04 ± 0.27 kPa T47D: 0.94 ± 0.25 kPa MDA‐MB‐231: 0.62 ± 0.21 kPa Detachment force 10 s: 2.21 ± 0.44 nN, 120 s: 13.47 ± 2.25 nN 10 s: 1.92 ± 0.48 nN, 120 s: 5.77 ± 0.56 nN 10 s: 1.71 ± 0.61 nN, 120 s: 4.60 ± 0.57 nN Across the three cell lines, greater reported invasive potential was associated with lower Young's modulus and lower cell–cell detachment force, with a strong positive correlation between stiffness and detachment force. F‐actin MDA‐MB‐231: localised in leading edges MCF‐7 and T47D: more evenly distributed	Omidvar et al. (2014), Iran
	• MCF‐10A (healthy) • MCF‐7 (tumorigenic, non‐invasive) • MDA‐MB‐231 (tumorigenic, invasive)	BioScope Catalyst, Japan Peak‐force AFM: pyramidal tip, ∼20 nm radius, 250 Hz; comparison with conventional 10‐μm‐diameter spherical probe, 1 Hz spherical‐probe indentation; Sneddon/Hertz‐type analysis according to probe	MCF‐10A: 0.7 ± 0.3 kPa (1 Hz), 250 ± 100 kPa (250 Hz) MCF‐7: 0.5 ± 0.1 kPa (1 Hz), 28 ± 12 kPa (250 Hz) MDA‐MB‐231: 0.3 ± 0.1 kPa (1 Hz), 25 ± 13 kPa (250 Hz) Values strongly dependent on measurement mode/loading rate; healthy cells more readily distinguished from tumourigenic cells at high‐frequency measurement F‐actin MCF10: denser and parallel MCF‐7 and MDA‐MB‐231: sparse and diffuse	Calzado‐Martín et al. (2016), Spain
	• HS68 – normal fibroblasts • MCF10A – normal fibrocystic mammary epithelium • MDA‐MB‐231 – metastatic breast cancer pleural effusion	Asylum Research, USA Cells associated with 3D isotropic silicon microstructures; soft V‐shaped cantilever, ∼0.02 N/m; 0.5 µm/s tip velocity; Hertz model, *ν* = 0.5	HS68: 1.86 ± 1.13 kPa MCF10A: 1.13 ± 0.84 kPa MDA‐MB‐231: 0.51 ± 0.35 kPa MCF‐10A significantly stiffer than MDA‐MB‐231	Nikkhah et al. (2010), USA
Bladder				
	Urothelium: • Non‐malignant: SV‐HUC‐1 • Malignant: MGH‐U1	JPK Nanowizard I, Germany Pyramidal Si_3_N_4_ probe (∼10 nm radius; *k* ≈ 0.01–0.02 N/m); live cells in culture medium at room temperature; loading up to 4 nN at 0.5 μm/s; Sneddon‐modified Hertz model	SV‐HUC‐1: 27.57 kPa MGH‐U1: 2.46 kPa Adhesion energy SV‐HUC‐1: 8.17 × 10^−16^ J MGH‐U1: 26.95 × 10^−16^ J MGH‐U1 cells had an ∼11‐fold lower effective elastic modulus than SV‐HUC‐1 cells. Cancer cells also showed greater adhesion energy and surface roughness	Canetta et al. (2014), UK
	Urothelium carcinoma • RT4 (grade I) • T24 (grade III)	BioScope Catalyst, USA Spherical borosilicate probe (radius ∼4.9 μm); live cells in fluid at room temperature; 500 pN trigger force with indentation <15% of cell height; Hertz/Sneddon‐based analysis	RT4: 0.47 ± 0.04 kPa T24: 0.21 ± 0.02 kPa High‐grade/invasive T24 cells were significantly softer than low‐grade/non‐invasive RT4 cells F‐actin: greater cytoplasmic actin density in RT4; T24 predominantly showed thin cortical actin with sparse, disorganised/fragmented cytoplasmic F‐actin	Liu et al. (2014), Canada
	Urothelial epithelial cells • Benign: Hu609, HCV29 • Malignant: Hu456, T24, BC3726	AFM (home‐built) Si_3_N_4_ probe, 20–30 nm tip radius; *k* = 0.03–0.1 N/m; 190 nm/s; force up to 20 nN; indentation up to ∼1.6 µm; cell centre; living cells in culture medium at room temperature; Sneddon/paraboloidal‐tip model, *ν* = 0	Hu609: 12.9 ± 4.8 kPa HCV29: 10.0 ± 4.6 kPa BC3726: 1.4 ± 0.7 kPa T24: 1.0 ± 0.5 kPa Hu456: 0.4 ± 0.3 kPa Non‐malignant cells were approximately one order of magnitude stiffer than malignant/transformed cells	Lekka et al. (1999), Poland
Prostate				
	Epithelium • Benign: BPH • Malignant: LNCaP, PC‐3	Veeco, UK V‐shaped Si_3_N_4_ cantilever (*k* ≈ 0.06 N/m); live cells in culture medium at room temperature; central‐cell indentation at 5.7 μm/s; pyramidal Hertz model fitted to ≤3 μm indentation	BPH: 2.797 ± 0.491 kPa LNCaP: 0.287 ± 0.052 kPa PC‐3: 1.401 ± 0.162 kPa BPH was stiffer than both malignant lines; however, highly invasive PC‐3 cells were stiffer than non‐invasive LNCaP cells, indicating that stiffness did not decrease monotonically with metastatic potential	Faria et al. (2008), UK
	Cell‐line experiment • Normal: PZ‐HPV‐7 • Metastatic PC‐3	JPK NanoWizard, Germany DNP‐S10 probe, (*k* = 0.06) N/m, 35° half‐angle; thermal‐noise calibration; 0.5, 1 and 2 nN loading forces; 2‐s relaxation; Hertz model	PZ‐HPV‐7: ∼2.20 kPa Metastatic PC‐3: ∼1–2 kPa Metastatic PC‐3 cells were softer and less viscous than normal PZ‐HPV‐7 cells and showed a more disorganised F‐actin architecture	Zeng et al. (2023), China
	Clinical tissue experiment • BPH • Low‐risk PCa • High‐risk PCa	Same AFM platform; fresh tissue; tissue measurements clustered into cellular, intermediate and non‐cellular/ECM components using K‐means and fuzzy C‐means	Whole‐tissue mean Young's modulus BPH: 1.853 ± 0.016 kPa Low‐risk PCa: 1.111 ± 0.0192 kPa High‐risk PCa: 0.676 ± 0.0145 kPa Cellular component BPH: 1.21 ± 0.38 kPa Low‐risk PCa: 0.817 ± 0.42 kPa High‐risk PCa: 0.594 ± 0.29 kPa Both whole‐tissue and cellular‐region stiffness showed a stepwise decrease from BPH to low‐risk to high‐risk PCa, whereas the non‐cellular/ECM components showed no significant viscoelastic differences	
Skin				
	Epidermal melanocytes: • Normal human epidermal melanocytes (NHEM) • Non‐invasive RGP melanoma: SBC12 Invasive VGP melanoma: WM115 Metastatic melanoma: WM239A	JPK NanoWizard II, Germany 5‐µm‐radius spherical probe; *k* ≈ 0.03 N/m; 1 µm/s; max force 500 pN; indentation ∼500–1000 nm over nuclear region; live cells at 37 °C; Hertz spherical model	On polystyrene after 24 h NHEM: 0.308 ± 0.018 kPa SBC12: 0.240 ± 0.012 kPa WM115: 0.147 ± 0.012 kPa WM239: 0.421 ± 0.030 kPa Stiffness decreased from normal melanocytes through RGP to VGP melanoma, but increased again in metastatic WM239A cells to above normal melanocytes, indicating non‐monotonic mechanical progression and increased stiffness plasticity	Weder et al. (2014), Switzerland
	Keratinocytes • Normal: HaCaT • Melanoma: WM1366 Samples studied • HaCaT • shSCR: Gal‐3 expressing WM1366 • shGal3: Gal‐3 silenced WM1366	Bruker MultiMode 8, USA Pyramidal Si_3_N_4_ tip; *k* ≈ 0.12 N/m; max force 10 nN; 2–3 µm/s; indentation ∼25% cell height over nuclear region; PFA‐fixed cells in PBS; Sneddon model	HaCaT: 31.9 ± 0.4 kPa shSCR: 21.8 ± 0.5 kPa shGal3: 16.1 ± 0.6 kPa RGP melanoma cells were softer than keratinocytes, further softening following Gal‐3 silencing. F‐actin was cytoplasmic in HaCaT but predominantly peripheral in melanoma cells	Herrera‐Reinoza et al. (2023), Brazil
Oral epithelium				
	SCC: • PE/CA‐PJ15 • PE/CA‐PJ15‐d (with higher degree of malignancy)	Assing S.p.A., Italy Fixed cells; 2.5‐µm SiO_2_ spherical probe; *k* ≈ 0.5 N/m; 0.01 µm/s; 30–40 nm indentation; Hertz model	PE/CA‐PJ15: 6.75 MPa PE/CA‐PJ15‐d: 4.36 MPa More malignant PE/CA‐PJ15‐d cells showed a 35.5% reduction in Young's modulus. The unusually high MPa‐scale values likely reflect the fixed‐cell preparation and very shallow 30–40 nm indentation	Lasalvia et al. (2015), Italy
	TSCC cells with differing metastatic potential • Lower potential: UM2, UM1/miR‐138 • Higher potential: UM1, UM1/Control	JPK NanoWizard II, Germany Live cells at 37 °C; 1.01‐µm flat‐ended cylindrical Si_3_N_4_ probe; *k* = 0.037 N/m; ∼3 µm indentation over nuclear region; rate‐jump method; *ν* = 0.5	UM2: 6.16 ± 1.39 kPa UM1/miR‐138: 6.83 ± 1.65 kPa UM1: 3.65 ± 1.01 kPa UM1/Control: 3.85 ± 0.85 kPa Higher metastatic potential was associated with lower Young's modulus, EMT and reduced/disorganised F‐actin and β‐tubulin	Zhou et al. (2013), China
	OSCC cell lines (SCC‐4, SCC‐9, SCC‐15)	Asylum Research, USA Live cells at 37 °C; 1.5–4 µm polystyrene‐bead probe; *k* ≈ 0.01 N/m; 500–1000 pN trigger force; 1 Hz; cell‐centre indentation; Hertz model	SCC‐4: 1.122 ± 0.148 kPa SCC‐9: 0.553 ± 0.059 kPa SCC‐15: 0.208 ± 0.029 kPa Lower stiffness across the three cell lines was associated with stronger EMT‐related and migratory phenotypes, with ↑ Snail2, N‐cadherin and vimentin and ↓ E‐cadherin. TGF‐β1‐induced EMT in SCC‐4 also resulted in significant mechanical softening	Park et al. (2016), South Korea
	Oral epithelial cells • Normal • OSCC: SCC4, SCC25, H400	BioScope Resolve, Japan PeakForce QNM; live cells in fluid; 65‐nm‐radius conical probe; *k* ≈ 0.12 N/m; 100 × 100 µm maps; DMT/JKR models, *ν* = 0.5	All models showed significantly lower modulus in pooled OSCC cells DMT: normal 164.25 kPa, OSCC 91.17 kPa JKR: normal 154.66 kPa, OSCC 35.39 kPa OSCC cells were significantly softer than normal oral keratinocytes across models. Cytoskeleton: disorganised cytoskeletal architecture with ↑ vimentin and β‐tubulin in OSCC cells Total deformation, elastic and plastic energies were significantly higher in OSCC cells than in normal keratinocytes	Guan et al. (2025), New Zealand
Kidney				
	Human renal cells • RC‐124–normal • A‐498–carcinoma ACHN‐adenocarcinoma	Asylum Research, USA Live cells; pyramidal Si_3_N_4_ tip, *k* ≈ 0.01 N/m; max force 3 nN; force‐volume mapping; Hertz analysis; ∼50 cells/cell line	RC‐124: 9.38 kPa A‐498: 7.41 kPa ACHN: 2.48 kPa A‐498 and ACHN 20% and 70%, respectively, softer than RC‐124	Rebelo et al. (2013), Brazil
Oesophagus				
	Normal oesophagus squamous cell line • EPC2 Barrett's oesophagus columnar cell lines • CP‐A: non‐dysplastic metaplasia CP‐D: high‐grade dysplasia	Asylum Research, USA Live cells in imaging buffer; sharp Si tip, radius ∼50 nm, *k* ≈ 0.01 N/m; 1 nN trigger force; 2 µm/s; 64 indentations over nuclear region; segmental Hertz analysis	EPC2: 4.7 kPa CP‐A: 3.1 kPa CP‐D: 2.6 kPa EPC2 is 1.5x and 1.8x stiffer than CP‐A and CP‐D, respectively Progressive softening occurred from normal → metaplastic → dysplastic cells	Fuhrmann et al. (2011), USA
Bone				
	Human osteosarcoma • HOS (non‐aggressive & non‐metastatic) • 143B (highly aggressive & metastatic)	Asylum Research, USA Live cells at 37 °C; 7.24‐µm silica spherical probe; *k* ≈ 0.126 N/m; thermal‐noise calibration; 1.5 nN peak force; ∼400–800 nm indentation; Hertz/Sneddon models; *ν* = 0.5	HOS: 1.54 ± 0.23 kPa 143B: 0.97 ± 0.05 kPa Metastatic 143B cells were ∼37% softer than non‐metastatic HOS cells; HOS also showed denser actin stress‐fibre networks	Yurtsever et al. (2021), Japan
Body‐cavity effusions				
	Metastatic tumour and benign mesothelial cells from body‐cavity effusions	Nanoscope IV Bioscope Sharp Si_3_N_4_ tip, ∼20 nm radius, *k* ≈ 0.02 N/m; central/nuclear‐region measurements; Hertz model, *ν* = 0.5; cytospin‐prepared patient effusion cells	Standard 12‐h ex vivo preparation Benign: 1.97 ± 0.70 kPa Malignant: 0.53 ± 0.10 kPa Cytospin preparation Benign: 2.53 ± 1.30 kPa Malignant: 0.38 ± 0.2 kPa Metastatic tumour cells were >80% softer than benign mesothelial cells and showed *a* > sixfold narrower stiffness distribution. Tumour cells were ∼33% less adhesive than benign mesothelial cells	Cross et al. (2008), USA

### 3.1 Breast

Breast cancer provides a particularly informative example of both the utility and limitations of Young's modulus as a cancer mechanophenotype. Under conventional two‐dimensional culture conditions, several AFM studies have reported lower apparent Young's modulus in breast cancer cell lines than in non‐tumourigenic MCF‐10A cells. MCF‐10A cells were approximately 1.4–2.5‐fold stiffer than MCF‐7 cells in independent studies (Li et al. 2008; Wang et al. 2016), while Nikkhah et al. (2010) similarly found MCF‐10A cells to be significantly stiffer than metastatic MDA‐MB‐231 cells. These studies therefore support a cellular‐softening phenotype in standard culture conditions, although the reported absolute modulus values vary considerably between experimental protocols.

The mechanical differences extend beyond elasticity alone. Wang et al. (2016) found MCF‐7 cells to exhibit both lower elasticity and lower viscosity than MCF‐10A cells, together with increased surface roughness and a more disorganised F‐actin architecture. Li et al. (2018) likewise identified lower Young's modulus and shorter relaxation times in MCF‐7 cells, indicating that malignant phenotype is associated with alterations in both elastic and time‐dependent viscoelastic behaviour. These observations support a multidimensional mechanical phenotype rather than stiffness alone as a discriminator of malignant state.

Cytoskeletal organisation provides one mechanistic basis for these differences. In MCF‐10A cells, well‐organised apical actin stress fibres corresponded to regions with apparent Young's modulus values up to an order of magnitude greater than neighbouring regions lacking these structures, whereas tumourigenic MCF‐7 and MDA‐MB‐231 cells contained few comparable apical stress fibres (Calzado‐Martín et al. 2016). Li et al. (2008), McEwen et al. (2013) and Wang et al. (2016) similarly associated reduced stiffness with altered and less organised actin architecture. Importantly, however, the relationship between cytoskeletal organisation and AFM‐derived stiffness is not fixed: Calzado‐Martín et al. (2016) demonstrated that increasing the probing frequency markedly amplified the stiffness difference between MCF‐10A and malignant cells while reducing the mechanical distinction between non‐invasive MCF‐7 and invasive MDA‐MB‐231 cells. Thus, the apparent association between Young's modulus and malignant potential depends partly on the timescale at which cellular mechanics are interrogated.

Mechanical differences among breast cancer cell lines also extended to cell–cell adhesion. Omidvar et al. (2014) compared the non‐invasive MCF‐7, slightly invasive T47D and highly invasive MDA‐MB‐231 cell lines. Across these distinct cell lines, greater reported invasive potential was associated with lower cell–cell detachment forces and lower Young's modulus. MDA‐MB‐231 cells exhibited the lowest stiffness and detachment force, whereas MCF‐7 cells showed the highest, producing a strong positive correlation between these parameters (Omidvar et al. 2014). These differences were accompanied by altered cadherin expression and redistribution of actin filaments, illustrating how elasticity and adhesion can change together in association with invasive phenotype rather than acting as independent mechanical biomarkers.

Crucially, however, reduced Young's modulus is not an invariant feature of breast cancer cells with greater reported invasive potential. Cellular geometry and mechanical context can substantially alter the measured phenotype. Liboz et al. (2023) found that unconstrained MDA‐MB‐231 cells were softer than MCF‐7 cells, whereas adhesive micropatterning increased both the modulus and dispersion of MDA‐MB‐231 cells sufficiently to reverse this relationship. These effects were associated with differences in cell spreading and cytoskeletal organisation, demonstrating that the same cell line can adopt different apparent mechanical states depending on its physical environment.

An even more striking example is provided by three‐dimensional invasion. Staunton et al. (2016) showed that MDA‐MB‐231 cells actively invading collagen‐I matrices became progressively stiffer with increasing matrix embeddedness despite the well‐established relative softness of this cell line under conventional two‐dimensional conditions. Fully embedded cells were also stiffer than non‐embedded cells, and inhibition of Rho‐associated kinase (ROCK) attenuated this invasion‐associated stiffening, implicating actomyosin contractility in the mechanical adaptation to the three‐dimensional matrix. Thus, metastatic competence should not be viewed as requiring persistent cellular softening; rather, invasive cells appear capable of mechanically adapting between compliant and more contractile states according to environmental demands.

This distinction is equally important when moving from isolated cells to tissues. In human breast tissue, Ansardamavandi et al. (2016) identified an approximately 50% reduction in the Young's modulus of the putative cellular regions of cancerous samples relative to healthy tissue. In contrast, the fibrous regions of malignant tissue showed only a modest increase in modulus that was not statistically significant. These findings illustrate that cellular and tissue mechanics cannot be treated interchangeably: a tumour may contain relatively compliant malignant cells, while its overall mechanical behaviour also reflects stromal composition, ECM organisation and spatial heterogeneity.

### 3.2 Bladder

AFM studies of bladder epithelial models have consistently demonstrated lower apparent Young's modulus in malignant than in non‐malignant cells when comparisons are made under matched experimental conditions. Canetta et al. (2014) reported that malignant MGH‐U1 cells had an effective elastic modulus approximately 12‐fold lower than non‐malignant SV‐HUC‐1 urothelial cells. MGH‐U1 cells also exhibited approximately 2.6‐fold greater probe–cell adhesion energy, together with smaller lateral dimensions, greater thickness and increased surface roughness, indicating that malignant phenotype was associated with coordinated changes in several nanomechanical and morphological parameters rather than stiffness alone. Earlier, Lekka et al. (1999) similarly found Young's moduli of approximately 7.5–12.9 kPa in Hu609, derived from non‐malignant ureter epithelium, and HCV29 cells, derived from non‐malignant bladder urothelium, compared with approximately 0.3–1.4 kPa in the malignant BC3726, T24 and Hu456 carcinoma lines, depending on the assumed Poisson ratio. Together, these studies support a pronounced softening phenotype in these bladder‐cell models while also illustrating that malignancy‐related mechanical changes extend beyond elasticity alone.

Mechanical differences have also been observed between bladder cancer cell lines derived from urothelial carcinomas of different histological grades. Liu et al. (2014) found that RT4 cells, derived from a low‐grade, non‐invasive tumour, had a significantly higher Young's modulus than T24 cells, derived from a high‐grade, invasive tumour (0.47 ± 0.04 vs. 0.21 ± 0.02 kPa), indicating lower stiffness in the higher‐grade phenotype within this experimental model. These differences were accompanied by changes in F‐actin organisation. RT4 cells displayed greater cytoplasmic actin density, whereas most T24 cells exhibited predominantly thin cortical actin, sparse cytoplasmic filaments and a more disorganised, fragmented F‐actin network. These findings are consistent with an association between cytoskeletal reorganisation and reduced cellular stiffness in the higher grade, more aggressive phenotype, although they do not establish a universal monotonic relationship between tumour grade and Young's modulus.

The relationship between cytoskeletal organisation and mechanical phenotype was also evident in the comparison of SV‐HUC‐1 and MGH‐U1 cells. Canetta et al. (2014) found that SV‐HUC‐1 and MGH‐U1 cells differed not only in elasticity but also in adhesion, roughness and overall cellular geometry. The malignant MGH‐U1 cells were approximately 1.5‐fold smaller, 1.7‐fold thicker and 1.4‐fold rougher than SV‐HUC‐1 cells, while also showing substantially lower effective modulus and higher adhesion energy. This combination of morphological, adhesive and elastic alterations supports the interpretation of bladder cancer as a multidimensional mechanophenotype rather than one defined solely by Young's modulus.

These studies also illustrate the methodological dependence of AFM‐derived Young's modulus. Canetta et al. (2014) identified MGH‐U1 as equivalent to the T24 bladder cancer line yet reported a median effective modulus of approximately 2.46 kPa, whereas Liu et al. (2014) measured approximately 0.21 kPa for T24 cells using a different AFM protocol. The studies differed substantially in probe geometry, applied force and indentation conditions: Canetta et al. (2014) used a sharp pyramidal probe with loading up to 4 nN, whereas Liu et al. (2014) used a ∼4.87‐μm‐radius spherical probe and a 500‐pN trigger force while limiting indentation to less than 15% of cell height. Thus, while the direction of within‐study mechanical differences is consistent, absolute modulus values should not be compared across studies without accounting for methodology.

### 3.3 Prostate

Prostate cancer studies illustrate both the frequently observed softening associated with malignant transformation and the limitations of interpreting Young's modulus as a monotonic marker of metastatic potential. Zeng et al. (2023) found that normal PZ‐HPV‐7 prostate epithelial cells exhibited an average Young's modulus of approximately 2.20 kPa, whereas metastatic PC‐3 cells showed a narrower distribution centred between approximately 1 and 2 kPa. PC‐3 cells also exhibited lower viscosity, consistent with a more compliant and fluid‐like mechanical phenotype. These differences were accompanied by marked cytoskeletal alterations: PZ‐HPV‐7 cells contained prominent, well‐organised F‐actin bundles aligned along the long axis of the cell, whereas PC‐3 cells displayed more diffusely distributed and disordered stress fibres, together with numerous membrane protrusions and increased surface irregularity (Zeng et al. 2023).

A similar reduction in stiffness was observed in clinical prostate tissue. Zeng et al. (2023) reported mean Young's moduli of approximately 1.85 kPa for benign prostatic hyperplasia (BPH) tissue, 1.11 kPa for low‐risk prostate cancer and 0.68 kPa for high‐risk disease. When the heterogeneous tissue measurements were separated by clustering, the cellular regions showed a corresponding decrease from 1.21 ± 0.38 kPa in BPH to 0.817 ± 0.42 kPa in low‐risk and 0.594 ± 0.29 kPa in high‐risk prostate cancer, whereas the viscoelastic properties of the non‐cellular regions did not differ significantly. These findings suggest that the reduction in tissue stiffness was predominantly associated with the cellular compartment rather than a uniform softening of all tissue components (Zeng et al. 2023).

However, the relationship between stiffness and metastatic potential was not reproduced uniformly across prostate cancer cell lines. Faria et al. (2008) reported apparent Young's moduli of 2.797 ± 0.491 kPa for BPH cells, 1.401 ± 0.162 kPa for highly invasive PC‐3 cells and only 0.287 ± 0.052 kPa for non‐invasive LNCaP cells. Thus, although both malignant lines were softer than the BPH cells, the more invasive PC‐3 cells were substantially stiffer than the non‐invasive LNCaP cells, contrary to the expectation that increasing metastatic potential would be associated with progressive cellular softening. The authors therefore concluded that different prostate cancer phenotypes could be mechanically distinguished but that Young's modulus did not show a simple inverse relationship with metastatic potential (Faria et al. 2008).

Zeng et al. (2023) further identified biochemical remodelling of the tumour microenvironment despite the absence of significant differences in the AFM‐derived viscoelasticity of the clustered non‐cellular regions. Collagen I expression was greatest in BPH tissue and decreased with increasing Gleason grade and metastasis, whereas matrix metalloproteinase‐2 (MMP‐2) expression was greater in prostate cancer and highest in metastatic specimens. The authors proposed that increased MMP‐2 expression may contribute to collagen degradation and thereby facilitate tumour‐cell migration and tissue softening (Zeng et al. 2023). Thus, prostate tumour mechanics appear to reflect interactions between cellular cytoskeletal organisation and extracellular‐matrix remodelling rather than a single, uniformly progressive reduction in Young's modulus.

### 3.4 Skin

Melanoma provides a clear example of non‐monotonic mechanical behaviour across malignant progression. Weder et al. (2014) compared normal human epidermal melanocytes with radial‐growth‐phase (RGP), vertical‐growth‐phase (VGP) and metastatic melanoma cells. RGP and VGP cells exhibited lower Young's modulus than normal melanocytes, with VGP cells showing the lowest stiffness. In contrast, metastatic WM239A cells were stiffer than both earlier‐stage melanoma cells and normal melanocytes under several adhesion conditions. Notably, the VGP WM115 and metastatic WM239A lines were derived from the primary tumour and a metastasis from the same patient, providing a lineage‐related comparison of later‐stage progression (Weder et al. 2014). The magnitude of this response varied substantially with the adhesion substrate, indicating that metastatic melanoma cells acquired an enhanced capacity to modulate their stiffness in response to extracellular cues.

A complementary study by Herrera‐Reinoza et al. (2023) examined the role of galectin‐3 (Gal‐3) in RGP WM1366 melanoma cells. HaCaT keratinocytes exhibited a higher apparent Young's modulus than Gal‐3‐expressing WM1366 cells (31.9 ± 0.4 vs. 21.8 ± 0.5 kPa), while silencing Gal‐3 reduced the modulus further to 16.1 ± 0.6 kPa. Because HaCaT keratinocytes are not a lineage‐matched melanocytic control, this comparison should not be interpreted as a direct normal‐melanocyte‐to‐melanoma progression model. Nevertheless, the within‐line manipulation demonstrates that reduced Gal‐3 expression is associated with increased deformability in WM1366 cells (Herrera‐Reinoza et al. 2023). Galectin‐3 is a matricellular protein involved in cell–cell and cell–matrix interactions, including adhesion and migration (Elola et al. 2007). In melanoma, reduced Gal‐3 expression has been associated with more advanced phenotypes and greater metastatic potential (Brown et al. 2012; Hayashi et al. 2019). The further reduction in stiffness following Gal‐3 silencing in WM1366 cells (Herrera‐Reinoza et al. 2023) is therefore consistent with an association between reduced Gal‐3 expression, altered cytoskeletal organisation and increased cellular deformability.

Cytoskeletal and morphological differences accompanied these mechanical changes. HaCaT keratinocytes displayed dense filamentous structures at cell–cell junctions, with F‐actin distributed throughout the cytoplasm, whereas WM1366 melanoma cells lacked these junctional filamentous structures and showed F‐actin concentrated predominantly around the plasma membrane (Herrera‐Reinoza et al. 2023). These observations further support a relationship between cytoskeletal organisation and the measured mechanical phenotype, although the comparison is between different epithelial and melanocytic lineages.

Taken together, these findings suggest that melanoma progression may involve not simply progressive softening but increasing mechanical plasticity. Weder et al. (2014) proposed that early softening may facilitate deformation, stromal invasion and intravasation, whereas metastatic cells may subsequently benefit from an enhanced ability to increase or decrease their stiffness in response to changing adhesion and extracellular‐matrix conditions. Such plasticity could facilitate adaptation during circulation, extravasation and colonisation of distant tissues. Importantly, the authors regarded this model as provisional because it was based on a limited number of melanoma cell lines rather than primary patient‐derived melanoma cells (Weder et al. 2014).

### 3.5 Oral Mucosa

Marked nanotopographic and nanomechanical differences are evident between normal oral epithelial cells and oral squamous cell carcinoma (OSCC). Normal oral keratinocytes display uniform, well‐defined contours, whereas OSCC cells exhibit irregular spindle, round or elliptical morphologies, poorly defined borders, prominent filopodia and protrusive surface structures and increased surface complexity on AFM imaging (Guan et al. 2025). Similar protrusive features were observed in more metastatic tongue squamous cell carcinoma (TSCC) cells, which demonstrated increased microvilli and filopodia‐like processes (Zhou et al. 2013).

Guan et al. (2025) reported significantly lower Young's modulus in OSCC cells than in normal keratinocytes across the mechanical models examined. Using a Poisson ratio of 0.5, median Young's modulus decreased from 164.25 kPa in normal cells to 91.17 kPa in OSCC cells using the DMT model, and from 154.66 to 35.39 kPa using the JKR model (both *p* < 0.05).

These mechanical and morphological differences were accompanied by substantial cytoskeletal remodelling (Guan et al. 2025; Zhou et al. 2013). Guan et al. (2025) reported increased but disorganised vimentin and β‐tubulin expression in OSCC cells relative to normal keratinocytes, with the fibres appearing asymmetric and disorganised. In contrast, comparisons among TSCC cells with different metastatic potential showed that cells with greater metastatic potential and lower elastic modulus had reduced intensity and organisation of both F‐actin and β‐tubulin (Zhou et al. 2013). These findings suggest that cytoskeletal changes depend on the biological comparison being made and the specific cytoskeletal component examined rather than reflecting a uniform loss or gain of cytoskeletal material during malignant progression.

Guan et al. (2025) further expanded mechanical profiling beyond Young's modulus by examining deformation‐energy parameters. OSCC cells demonstrated significantly greater total deformation, elastic and plastic energies than normal cells, together with higher adhesion forces. These findings indicate that malignant transformation alters not only cellular stiffness but also the amount and partitioning of mechanical energy associated with recoverable and non‐recoverable deformation. Such parameters may therefore provide complementary information to Young's modulus within a broader mechanophenotypic profile.

Mechanical softening was also associated with an EMT‐related phenotype. Park et al. (2016) compared three distinct OSCC cell lines: SCC‐4, SCC‐9 and SCC‐15. SCC‐4 exhibited a predominantly cuboidal epithelial morphology, whereas SCC‐9 and SCC‐15 displayed more spindle‐shaped mesenchymal morphology. Across these cell lines, increasing expression of Snail2, N‐cadherin and vimentin, reduced E‐cadherin expression and enhanced migration were observed in the same order as decreasing mechanical stiffness, with SCC‐4 showing the highest stiffness, SCC‐9 an intermediate stiffness and SCC‐15 the lowest (Park et al. 2016). These findings indicate an association between a stronger EMT/migratory phenotype and mechanical softening. This association was further supported within a single cell line as TGF‐β1‐induced EMT in SCC‐4 cells produced corresponding EMT‐marker changes and significant mechanical softening (Park et al. 2016). Similarly, Zhou et al. (2013) found that more metastatic TSCC cells exhibited higher vimentin and lower E‐cadherin expression, together with reduced elastic modulus.

Overall, the oral cancer studies reviewed here consistently reported mechanical softening in malignant or more aggressive phenotypes under their respective experimental conditions. Guan et al. (2025) demonstrated significantly lower Young's modulus in OSCC cells than in normal oral keratinocytes, while Lasalvia et al. (2015) reported a 35.5% reduction in the more malignant PE/CA‐PJ15‐d phenotype relative to PE/CA‐PJ15. Park et al. (2016) demonstrated decreasing stiffness across SCC‐4, SCC‐9 and SCC‐15 in association with stronger EMT‐related and migratory phenotypes, and Zhou et al. (2013) similarly found lower elastic modulus in TSCC cells with greater metastatic potential. These directional similarities should nevertheless be interpreted within their methodological context because the studies differed substantially in sample preparation, probe geometry, indentation protocol and mechanical model.

### 3.6 Other Models (Kidney, Oesophagus, Bone and Body‐Cavity Effusions)

Across diverse tumour models, AFM‐based analyses frequently demonstrate reduced cellular stiffness with neoplastic transformation or greater metastatic potential, although the magnitude and direction of change depend on the biological context and material being measured. In kidney epithelial cells, median Young's modulus decreased from non‐tumourigenic RC‐124 cells (9.38 kPa) to A‐498 carcinoma cells (7.41 kPa) and ACHN adenocarcinoma cells (2.48 kPa), corresponding to approximately 21% and 74% lower stiffness, respectively, relative to RC‐124 cells (Rebelo et al. 2013). These differences represent distinct tumour phenotypes rather than sequential stages of malignant progression.

A similar reduction in stiffness was observed in a model of pre‐neoplastic oesophageal progression, in which normal EPC2 squamous cells exhibited the greatest Young's modulus (∼4.7 kPa), followed by metaplastic CP‐A (∼3.1 kPa) and dysplastic CP‐D cells (∼2.6 kPa) (Fuhrmann et al. 2011). In patient‐derived body‐cavity effusions, metastatic tumour cells were more than 80% softer than benign reactive mesothelial cells and exhibited a Young's modulus distribution more than six times narrower, demonstrating a substantially narrower range of measured mechanical phenotypes within the metastatic tumour‐cell population (Cross et al. 2008). The clinical cohort included metastatic carcinomas of lung, breast and pancreatic origin, although the mechanical results were analysed collectively rather than as tumour‐specific phenotypes. Taken together, these findings support reduced Young's modulus as a recurrent, but not universal, feature of malignant and premalignant models examined.

AFM stiffness nanotomography of oesophageal cells further revealed depth‐dependent variation in Young's modulus, highlighting the layered mechanical architecture of individual cells. Although all three cell types exhibited heterogeneous stiffness profiles, metaplastic and dysplastic cells showed progressively more frequent non‐monotonic ‘breakthrough' events—sawtooth‐like discontinuities in force–distance curves in which cellular resistance to the AFM tip suddenly decreased. These events were most frequent in dysplastic CP‐D cells, intermediate in metaplastic CP‐A cells and least frequent in normal EPC2 cells. Fuhrmann et al. (2011) proposed that these events represent local failure of prestressed cytoskeletal structures, whereby stresses applied to cortical structures are transmitted through intermediate filaments to deeper stress fibres that subsequently fail, allowing superficial material to be displaced through the disrupted filamentous network. The increased frequency of such events with premalignant progression suggests that greater compliance may reflect reduced cytoskeletal structural integrity rather than simply greater elastic bending.

Yurtsever et al. (2021) extended mechanical profiling to osteosarcoma cells and their exosomes, nanoscale extracellular vesicles involved in intercellular communication. Highly metastatic 143B cells were approximately 37% softer than non‐metastatic HOS cells (0.97 ± 0.05 vs. 1.54 ± 0.23 kPa), consistent with greater deformability in the more aggressive phenotype. In contrast, exosomes derived from 143B cells exhibited a substantially higher Young's modulus than those derived from HOS cells (∼192 vs. ∼118 MPa), representing an increase of approximately 63%. Proteomic analyses showed enrichment of elastic‐fibre‐associated proteins in 143B‐derived exosomes. On this basis, the authors proposed that metastatic cells may release stiffness‐associated proteins through exosomes, thereby contributing to preservation of a softer parental‐cell phenotype (Yurtsever et al. 2021). The markedly different mechanics of metastatic and non‐metastatic tumour‐derived exosomes, together with their accessibility in bodily fluids, suggest that exosome nanomechanics may warrant investigation as a potential non‐invasive biomarker.

## 4 Discussion

The evidence reviewed here supports altered cellular mechanics as a recurrent feature of carcinogenesis but challenges an overly simplified model in which increasing malignancy is invariably accompanied by progressive cellular softening. Several studies demonstrate mechanical differences across cells or pathological states with differing malignant, invasive or histological phenotypes (Fuhrmann et al. 2011; Liu et al. 2014; Zeng et al. 2023). Notably, Fuhrmann et al. (2011) demonstrated graded reductions in Young's modulus across normal, metaplastic and dysplastic oesophageal cells, providing evidence that nanomechanical alterations can arise before invasive malignancy. This finding is particularly relevant to early detection as AFM‐based mechanophenotyping could potentially identify biomechanical abnormalities during premalignant transformation before invasive malignancy is established. In principle, such graded alterations could enable the stratification of cells along a biomechanical risk spectrum. However, the establishment of clinically meaningful mechanical thresholds will require considerably greater methodological standardisation and prospective validation than is currently available.

Although cellular softening is frequently observed during malignant transformation, mechanical behaviour is neither static nor universally monotonic. Instead, the available evidence suggests that cancer cells exhibit mechanical plasticity, reflecting their capacity to reorganise the cytoskeleton and modify their mechanical state according to the disease stage and environmental demands (Liboz et al. 2023; Staunton et al. 2016; Weder et al. 2014). Mechanical softening may increase deformability and facilitate passage through confined extracellular spaces, whereas invasion‐associated stiffening may reflect increased actomyosin contractility or adaptation to changing ECM conditions (Fischer et al. 2020; Staunton et al. 2016; Suresh 2007). For example, MDA‐MB‐231 breast cancer cells stiffened progressively during invasion into collagen‐I matrices (Staunton et al. 2016), whereas metastatic melanoma cells could exhibit greater stiffness than normal melanocytes and showed marked stiffness plasticity in response to different adhesion environments (Weder et al. 2014). Similarly, prostate cancer‐cell stiffness does not show a simple inverse relationship with invasive potential: Faria et al. (2008) found highly invasive PC‐3 cells to be substantially stiffer than non‐invasive LNCaP cells. These apparently divergent observations are not necessarily contradictory; rather, they indicate that cellular mechanics are influenced by tumour type, metastatic state, ECM composition and stiffness, cell geometry and confinement and adaptation to mechanical cues (Berger et al. 2020; Fischer et al. 2020; Liboz et al. 2023; Staunton et al. 2016; Weder et al. 2014). Young's modulus should therefore be interpreted as a context‐dependent mechanobiological parameter rather than a fixed material property or universally directional cancer biomarker.

An important distinction must also be made between mechanics measured at the single‐cell and tissue levels. Although individual malignant cells frequently exhibit reduced apparent Young's modulus under defined in vitro conditions, tumour tissues may become mechanically stiffer through ECM remodelling, increased collagen deposition and crosslinking, stromal activation and altered tissue architecture (Butcher et al. 2009; Levental et al. 2009; Paszek et al. 2005). In breast tumour models, for example, progressive tissue stiffening is accompanied by increased fibrillar collagen, collagen linearisation and crosslinking, whereas AFM measurements of human breast tissue have identified comparatively compliant cellular regions within malignant samples (Ansardamavandi et al. 2016; Levental et al. 2009). Cellular softening and tumour tissue stiffening are therefore not contradictory; they reflect distinct biological processes operating at different spatial scales. Moreover, a stiffened tumour microenvironment can itself influence cellular behaviour and mechanics through integrin‐mediated mechanotransduction, ROCK‐dependent cytoskeletal tension and focal‐adhesion signalling (Butcher et al. 2009; Levental et al. 2009; Paszek et al. 2005). Accordingly, AFM‐derived Young's modulus measured from an isolated cell should not be directly equated with the mechanical properties of an entire tumour.

Collectively, these findings suggest that the diagnostic significance of cellular mechanics may lie less in a universal Young's modulus threshold than in disease‐ and context‐specific mechanical phenotypes (Faria et al. 2008; Weder et al. 2014; Wu et al. 2018). A single measurement of stiffness may not adequately capture the heterogeneity and adaptive mechanical behaviour associated with carcinogenesis, particularly because cellular mechanics can vary with disease state, measurement conditions, cellular geometry and the surrounding mechanical environment (Liboz et al. 2023; Weder et al. 2014; Wu et al. 2018). Instead, mechanical differences across pathological states and malignant phenotypes may provide more meaningful information, as suggested by oesophageal, prostate and melanoma models (Fuhrmann et al. 2011; Weder et al. 2014; Zeng et al. 2023). The objective of AFM‐based early cancer detection may therefore be less about defining a single stiffness value that identifies malignancy across all tissues and more about establishing reproducible, tissue‐specific mechanical phenotypes across disease stages. In this context, Young's modulus may be most valuable as one component of a broader mechanophenotypic profile incorporating complementary parameters such as viscoelasticity, adhesion, deformation energy and nanotopography rather than as an isolated universal biomarker (Canetta et al. 2014; Guan et al. 2025; Omidvar et al. 2014; Wang et al. 2016).

## 5 Challenges and Future Directions

For such disease‐ and context‐specific mechanical phenotypes to become clinically useful, several methodological and translational challenges must first be addressed. A fundamental limitation is methodological variability. Probe geometry, cantilever calibration, indentation depth, loading rate, cellular measurement region, sample preparation, substrate conditions and contact‐mechanics model used to analyse force–distance curves can all influence the apparent Young's modulus (Gavara 2017; Guan et al. 2025; Wu et al. 2018; Zemła et al. 2018). Comparative measurements of the same cell type using different mechanical techniques and AFM configurations have demonstrated variations in measured modulus spanning several orders of magnitude, illustrating the difficulty of directly comparing absolute values obtained under different experimental conditions (Wu et al. 2018). Standardised acquisition, analysis and reporting protocols will therefore be essential for establishing reproducible mechanical reference ranges and determining whether clinically meaningful thresholds can be defined.

Biological heterogeneity presents an additional challenge. Cellular stiffness varies spatially and is influenced by cytoskeletal organisation, disease stage, cell geometry, ECM composition and adaptation to mechanical stress (Liboz et al. 2023; Weder et al. 2014; Zeng et al. 2023). Clinical specimens introduce further complexity because malignant cells may coexist with non‐malignant epithelial, stromal, inflammatory or other host‐cell populations with distinct mechanical phenotypes. Cross et al. (2007), for example, analysed metastatic adenocarcinoma cells alongside benign reactive mesothelial cells within clinical pleural effusions, while Sokolov et al. (2018) emphasised that cells recovered from the urine of patients with bladder cancer are not necessarily themselves malignant. Furthermore, experimental manipulation of cellular geometry and microenvironment can alter the measured mechanical phenotype, as demonstrated by changes in stiffness with adhesive confinement, extracellular‐matrix invasion and substrate conditions (Liboz et al. 2023; Staunton et al. 2016; Weder et al. 2014). These considerations reinforce the need to interpret Young's modulus as a context‐dependent mechanical phenotype rather than a fixed material property or universal cancer threshold.

Low measurement throughput and reliance on operator input have represented additional barriers to clinical translation; however, recent technological developments indicate that these limitations are increasingly addressable (Thomas‐Chemin et al. 2025; Xiao et al. 2024). Thomas‐Chemin et al. (2025) highlighted an important distinction between increasing the number of force curves acquired from a small number of cells and achieving true cellular high throughput by increasing the number of independent cells analysed. They therefore conceptualised cellular high‐throughput AFM in terms of analysing large numbers of individual cells rather than merely generating large numbers of measurements from a few cells. Automation of cell localisation, probe positioning, calibration, force acquisition and subsequent data processing may enable substantially larger cellular populations to be mechanically characterised while reducing dependence on manual intervention (Thomas‐Chemin et al. 2025). Artificial intelligence and machine‐learning approaches may further facilitate automated recognition of cellular targets and classification of large AFM datasets.

Recent experimental developments demonstrate the feasibility of this approach. Xiao et al. (2024) combined AFM force spectroscopy with deep‐learning‐based optical image recognition to automatically identify cellular nuclei and the AFM probe from bright‐field images, enabling automated positioning of both the sample stage and the probe for sequential single‐cell measurements. The system was applied directly to Young's modulus measurement and analysed 100 cells for each cell type and experimental condition. The method therefore addresses two important limitations of conventional AFM simultaneously: measurement throughput and dependence on manual cell localisation and probe positioning (Xiao et al. 2024). More broadly, advances in AFM automation, computer vision and artificial intelligence indicate that low throughput and operator dependence should not be regarded as immutable limitations of the technique, although they remain important practical barriers to large‐scale and clinical implementation (Thomas‐Chemin et al. 2025; Xiao et al. 2024).

Beyond improving throughput, these technological developments create opportunities to move beyond Young's modulus as an isolated parameter. AFM can provide complementary information on viscoelasticity, adhesion, deformation energy, spatial mechanical heterogeneity and surface nanotopography, potentially enabling multidimensional mechanophenotyping rather than reliance on a single stiffness measurement (Canetta et al. 2014; Guan et al. 2025; Omidvar et al. 2014; Wang et al. 2016). Recent advances in AFM nanomechanical mapping enable spatially resolved measurement of multiple mechanical parameters across a sample, including elastic modulus, adhesion and viscoelastic properties, thereby extending analysis beyond a single whole‐cell stiffness value (Garcia and Tejedor 2025). Such multiparametric approaches can characterise mechanical heterogeneity across individual cells, while advances in automated data processing and machine learning provide methods for integrating these increasingly complex datasets (Garcia and Tejedor 2025; Thomas‐Chemin et al. 2025).

In a clinically derived example, Sokolov et al. (2018) combined nanoscale AFM imaging of fixed, urine‐derived cells with machine‐learning analysis of surface parameters including roughness, directionality and fractal characteristics to distinguish samples from patients with and without bladder cancer. Their study achieved 94% diagnostic accuracy using five cells per urine specimen in a cohort of 43 controls and 25 patients with bladder cancer, although the method assessed prepared cell‐surface phenotypes rather than Young's modulus in living cells (Sokolov et al. 2018). These findings nevertheless illustrate the potential transition from unidimensional stiffness measurements towards multidimensional AFM‐derived physical phenotypes. Integration of AFM with complementary imaging or spectroscopic techniques may further strengthen biological interpretation and diagnostic assessment (Canetta et al. 2014; Guan et al. 2025).

Improved measurement efficiency and analytical sophistication, however, do not by themselves establish clinical utility. Much of the evidence for AFM‐derived Young's modulus as a cancer biomarker remains based on established cell lines examined under controlled in vitro conditions. Proof‐of‐concept studies using clinically derived specimens have demonstrated the potential of AFM‐based physical phenotyping. Cross et al. (2007), for example, identified substantial differences in Young's modulus between pathologically characterised metastatic adenocarcinoma cells and benign mesothelial cells from patient pleural effusions. Similarly, Sokolov et al. (2018) used machine‐learning analysis of AFM‐derived surface phenotypes to distinguish urine samples from patients with and without bladder cancer. These studies are encouraging but remain insufficient to establish generalisable diagnostic performance.

Translation into clinical practice will require prospective validation in larger, independent and clinically representative cohorts, together with comparison against established diagnostic reference standards. Critical questions include whether malignant or premalignant phenotypes can be reliably identified within heterogeneous clinical samples, whether measurements are reproducible across operators, instruments and laboratories and whether AFM‐derived mechanical phenotypes provide sufficient sensitivity, specificity and incremental diagnostic value to influence clinical decision‐making. At present, AFM‐derived Young's modulus should therefore be considered a candidate for an adjunctive biomechanical biomarker rather than an established cancer screening test.

## 6 Conclusion

AFM studies across diverse cancer models demonstrate that malignant transformation is frequently accompanied by substantial alterations in cellular mechanics, with reduced Young's modulus representing a common but not universal phenotype. The accumulated evidence indicates that cancer mechanics are better understood as a dynamic and heterogeneous mechanophenotype rather than as a simple transition from stiff normal cells to soft malignant cells. Disease stage, cytoskeletal organisation, cellular region, microenvironment and measurement methodology all influence the apparent mechanical state. The potential diagnostic value of Young's modulus is therefore unlikely to reside in a universal cut‐off. Instead, stiffness should be integrated with complementary parameters, including spatial mechanical heterogeneity, viscoelasticity, adhesion, deformation energy and nanotopography. Advances in automation, high‐throughput force acquisition and artificial intelligence are progressively mitigating some of the traditional limitations of cellular AFM and may enable mechanical characterisation across substantially larger cell populations. The next phase of the field should therefore move beyond demonstrating mechanical differences between normal and cancer cells towards establishing standardised, reproducible and clinically validated multidimensional mechanophenotypes in patient‐derived specimens.

## Funding

The authors have nothing to report.

## Conflicts of Interest

The authors declare no conflicts of interest.

## Data Availability

No new data were created or analysed in this study. Data sharing is not applicable to this article.
